# Short-Term Dietary Exposure to Ochratoxin A, Zearalenone or Fumonisins in Broiler Chickens: Effects on Cytochrome P450 Enzymes, Drug Transporters and Antioxidant Defence Systems

**DOI:** 10.3390/foods14244249

**Published:** 2025-12-10

**Authors:** Neenu Amminikutty, Matteo Cuccato, Watanya Jarriyawattanachaikul, Marta Gariglio, Donato Greco, Vito D’Ascanio, Giuseppina Avantaggiato, Achille Schiavone, Carlo Nebbia, Flavia Girolami

**Affiliations:** 1 Department of Veterinary Sciences, University of Turin, 10095 Grugliasco, Italy; neenu.amminikutty@unito.it (N.A.); matteo.cuccato@unito.it (M.C.); w.jarriyawat@gmail.com (W.J.); marta.gariglio@unito.it (M.G.); achille.schiavone@unito.it (A.S.); flavia.girolami@unito.it (F.G.); 2 Institute of Sciences of Food Production, Italian National Research Council, 70126 Bari, Italy; donato.greco@cnr.it (D.G.); vito.dascanio@cnr.it (V.D.); giuseppina.avantaggiato@cnr.it (G.A.)

**Keywords:** mycotoxins, oxidative stress, liver, chicken, drug-metabolizing enzymes

## Abstract

Ochratoxin A (OTA), Zearalenone (ZEA), and Fumonisins (FB) are common contaminants of poultry feed associated with oxidative damage and potentially dangerous residues in products from exposed animals. We investigated the molecular effects in broilers of a short-term (10 days) dietary exposure to OTA (0.26 mg/kg), ZEA (2.9 mg/kg), or FB (60 mg/kg) on cytochrome P450 enzymes (CYP), drug transporters (DT) and the antioxidant defence system. OTA markedly decreased serum antioxidant capacity, while all mycotoxins depressed reduced glutathione content and increased lipid peroxidation in the liver, indicating a hepatic pro-oxidant effect. All the tested mycotoxins also reduced both the activities and the gene expression of selected antioxidant enzymes in the liver and duodenum as a result of the modulation of the Nrf2/Keap1 pathway. Moreover, mycotoxins differentially altered the hepatic and intestinal gene expression of CYP enzymes (i.e., *CYP2A6*, *CYP2C45*, *CYP3A4*, and *CYP1A* isoforms). Finally, the transcription of selected DT (i.e., *ABCB1*, *ABCC2* and *ABCG2*) was generally enhanced in both the liver and duodenum. In conclusion, short-term exposure to OTA, ZEA, or FB at dietary concentrations higher than those recommended in the EU, but occurring in third countries, not only disrupt the antioxidant defence but also affect the expression of CYP and DT, which might potentially alter the kinetics of drugs and toxicants. Our results provide new insights into mycotoxin adverse effects in the light to assess the effectiveness of new mitigation strategies that contribute to food and feed safety.

## 1. Introduction

Poultry feed contamination by mycotoxins (MYC) poses a significant challenge to animal health and productivity, as well as to global food safety [1,2]. Ochratoxin A (OTA), Zearalenone (ZEA), and Fumonisins (FB) are among the most concerning MYC [3,4].

Ochratoxins, primarily produced by *Penicillium* and *Aspergillus* fungi [5], are among the most prevalent MYC found in poultry feed, with OTA being the most common one [6]. Although in chickens OTA exhibits a higher excretion rate and a lower bioavailability compared to mammalian species [7], the exposure to OTA-contaminated feed may result in significant liver and kidney damage, oxidative stress (OS), alterations in blood parameters, and immune suppression, ultimately impairing zootechnical performances [3,8,9].

ZEA, a *Fusarium* toxin, is known for its ability to bind oestrogen receptors due to the structural similarity with the endogenous agonist 17β-oestradiol, causing reproductive toxicity in several animal species and in humans [10]. Chickens are much less susceptible than mammalian species to such effects, which are reported to occur at ZEA concentrations far above those commonly detected under field conditions [11]. However, the repeated exposure of broilers to even moderate ZEA levels (2 mg/kg feed for 42 days) was recently shown to negatively affect growth performances and, in some cases, induce hepatic oxidative damage [12].

Other important MYC are FB, namely Fumonisin B1 (FB1), Fumonisin B2 (FB2) and Fumonisin B3 (FB3), with the mode of action mainly consisting in lipid peroxidation and disruption of ceramide and sphingolipid metabolism [13]. Avian species are relatively resistant to FB adverse effects, due to a very limited oral bioavailability and a rapid excretion [14]. Given that FB1 is the most prevalent toxic analogue, toxicological assessments of FB have been primarily focussed on this specific compound [15]. FB-contaminated feed has shown to affect enteric morphology, liver function, and overall growth performances in broilers [16,17].

As mentioned above, although with different mechanisms, most MYC, including OTA, ZEA, and FB, are able to alter cellular homeostasis in all animal species, triggering the generation of reactive oxygen and other radical species, as well as the imbalance of the antioxidant defence system [18].

Cytochrome P450 (CYPs) and other biotransformation enzymes represent key factors in the metabolic fate of drugs, toxins, and endogenous substrates. Little is known, particularly in avian species, about the interrelationships between CYPs and MYC [19]. For instance, the condition of being a substrate is usually considered a pre-requisite for enzyme induction [20]. Among the three selected MYC, the participation of CYPs in the generation of oxidated derivatives has been demonstrated so far for OTA [21,22] and ZEA [23], while only circumstantial evidence has been provided for FB [24]. Interestingly, the repeated administration of FB-contaminated diets to broiler chicks has been recently reported to increase *CYP1A4* mRNA at enteric level, pointing to a contribution of this CYP in FB metabolic fate [25]. Conversely, despite the implication of liver CYP1A5 in T-2 toxin hydroxylation [26], a down-regulation of such CYP isoform was reported in T2-toxin chronically treated chickens [27].

Drug transporters (DT), also referred to as efflux proteins, are important determinants of drug and toxicant kinetics, since they are known to limit the enteric absorption and favour the elimination of several foreign compounds, including MYC [28]. Scant information is available on both the expression of DT in poultry tissues and the possible interactions on DT expression driven by MYC in avian species [29]. Aflatoxin B1 (AFB1), a likely ABCG2 substrate [30], was found to upregulate the expression of both *ABCG2* (about two-fold) and *ABCC2* (about six-fold) in the duodenum but not in the liver from broiler chicks dietary exposed to very low AFB1 levels for a short period [31]. In the study by Antonissen et al. (2017) mentioned above, the effects of the repeated administration of FB were tested also on liver and enteric MDR1 (ABCB1) and MRP2 (ABCC2); only jejunal ABCC2 was affected [25]. It is important to remark that alterations in the expression and the activity of CYPs and/or DT have the potential to affect the kinetics and hence the persistence of drugs and toxicants in animal products [32].

In the frame of a project aimed at investigating the efficacy of novel organic compounds to limit the uptake and mitigate the toxicity of MYC, the present study evaluates the comparative toxicological effects of OTA, ZEA, or FB in broiler chickens subjected to a short-term (10 days) administration at dietary levels 2- to 3-fold higher than the recommended EU limits in feed. These concentrations occasionally occur in Europe but are not infrequent in many extra-European countries [33,34]. For example, in a survey concerning the worldwide occurrence of MYC in feed commodities, OTA concentrations higher than 0.25 mg/kg were detected at the 75th percentile in maize samples from South American countries, and FB levels between 40 and 50 mg/kg were recovered in maize from countries belonging to South America, South East Asia, and Oceania [2]. The main aim of our study was to gain further insight into MYC effects on OS and the antioxidant defence system, as well as on less investigated endpoints, such as the modulation of CYP enzymes and drug transporters, in order to evaluate in future studies the efficacy of novel MYC detoxifying agents (MYDA).

## 2. Materials and Methods

### 2.1. Preparation of Contaminated Diets and MYC Analysis

MYC-contaminated diets were prepared by spiking a basal feed with fungal culture extracts obtained from fungal strains provided by the ITEM Microbial Culture Collection of CNR-ISPA (https://item.bio-aware.com/, accessed on 9 December 2025), with accession numbers ITEM 126 (*Fusarium graminearum*), ITEM 9337 (*F. verticillioides*), and ITEM 4211 (*Aspergillus ochraceus*) for the production of ZEA, FB, and OTA, respectively. Fungal culture extracts containing ZEA, FB, or OTA were prepared by inoculating wheat (ZEA), maize (FB), and rice (OTA) substrates with each toxigenic strain. After a one-month incubation in the darkness at 25 °C, the inoculated substrates were oven-dried at 60 °C (55 °C for the inoculated rice), ground, and extracted with mixtures of water and organic solvents. In particular, 40 g *F. graminearum* culture was extracted with 100 mL of acetonitrile/water (90:10, *v*/*v*), 20 g of *F. verticillioides* culture was extracted with 100 mL of water/acetonitrile/methanol (50:25:25, *v*/*v*/*v*), and 30 g of *A. ochraceus* culture was extracted with 120 mL of water/acetonitrile (40:60, *v*/*v*).

The content of ZEA, FB, and OTA in each culture extract was determined by direct injection into an HPLC–UV/FLD system after appropriate dilution (1:500) with Milli-Q^®^ water (Milli-Q Direct 8 system, Waters Corporation, Milford, MA, USA) and subsequent filtration. The MYC concentrations (mean ± SD) in each fungal culture extract were obtained from three independent analyses. The amount of ZEA detected in the *F. graminearum* fungal extract was 1070 ± 16 µg/mL. The concentration of OTA in the *A. ochraceus* fungal extract was 86 ± 1 µg/mL. Mean concentrations of FB1 and FB2 in the extract were 10.1 ± 0.3 mg/mL and 5.7 ± 0.2 mg/mL, respectively. FB3 was also detected in the fungal extract at 3.8 ± 0.3 mg/mL.

Based on the estimated feed requirements for the entire trial and for each experimental group, approximately 20 kg of ZEA, FB, and OTA-contaminated diets were prepared. To this end, 1 kg of blank maize was artificially contaminated with a volume of fungal culture extract containing a known concentration of each MYC. This 1 kg batch of contaminated maize was then used to inoculate each 20 kg lot of feed, followed by thorough mixing to ensure homogeneous contamination. In particular, we used 56 mL of *F. graminearum* extract, corresponding to 60 mg ZEA; 70 mL of the *F. verticillioides* culture, corresponding to 704 mg FB1; 396 mg FB2 and 264 mg FB3; and 70 mL of the *A. ochraceus* extract, corresponding to 6 mg of OTA. The fortified maize was stored overnight at room temperature under a chemical fume hood to allow solvent evaporation and to ensure its complete removal. It was then milled and homogenized and finally incorporated into the basal diet (20 kg). After mixing, five (5) subsamples from each artificially contaminated feed were collected and analyzed to determine the exact MYC concentration (mean ± SD, *n* = 5) and to assess homogeneity across the 20 kg batch. MYC analysis was performed using officially validated analytical methods. In particular, ZEA was analyzed according to the ISO 17372:2008 [35]. For FB analysis, the AOAC Official Method 2001.04 was used. OTA was determined following the standard method-EN 16007:2009 [36].

The resulting OTA concentration in the final diet averaged 0.26 ± 0.02 mg/kg (RSD = 6%). The ZEA contaminated diet showed a toxin concentration of 2.9 ± 0.2 mg/kg (RSD = 7%). FB concentrations in the diet were 33 ± 3 mg/kg (FB1), 17 ± 2 mg/kg (FB2), and 11 ± 2 mg/kg (FB3) (mean ± SD; *n* = 5), with RSD below 20%. The low RSD values confirmed the homogeneity of the diets with respect to MYC distribution.

To exclude any co-contamination with other MYC, the basal diet and the MYC-contaminated diets were analyzed according to the multi-MYC method developed by Solfrizzo et al. [37]. Except for FB, the basal diet did not show any detectable MYC contamination. The basal diet contained 1.3 ± 0.2 mg/kg of FB1, 0.4 ± 0.1 mg/kg of FB2, and 0.3 ± 0.4 mg/kg of FB3 (mean ± SD, *n* = 5).

### 2.2. LC-FLD/PDA/MS Analysis of MYC

ZEA analysis was performed using the ACQUITY UPLC™ system (Waters Corporation, Milford, MA, USA) combined with a PDA detector (PDA ACQUITY UPLC^®^) (Waters Corporation) as described by Ragoubi et al. [38]. OTA analysis was performed using an HPLC Agilent series 1100 (Agilent, Santa Clara, CA 95051, USA). OTA elution was achieved using a Simmetry Shield analytical column 150 mm × 4.6 mm, 5 μm particles (Waters Corporation). The column was preceded by a KrudKatcher HPLC In-Line Filter 2.0 μm (Phenomenex, Torrance, CA, USA). During the analysis, the column temperature was maintained at 35 °C. The injection volume was 20 μL. The flow rate of the mobile phase was 0.250 mL/min. OTA was eluted under isocratic conditions using a mobile phase composed of acetonitrile/water (60:40 *v*/*v*) and containing 1% acetic acid. For the analytical detection of OTA, the fluorimetric detector was set at wavelengths of 333 nm (λex) and 440 nm (λem). OTA was eluted after 4.7 min. Data acquisition and instrument control were performed using the Chemstation software (Agilent). FB were analyzed using the ACQUITY UPLC™ system (Waters Corporation) coupled with a mass detector (ACQUITY QDa). The separation of MYC was achieved using a Cortecs^®^ UPLC C18 analytical column (150 mm × 2.1 mm, 2.7 μm) (Waters Corporation), preceded by a Van-Guard^®^ Cartridge pre-column (2.1 mm × 5 mm) (Waters Corporation). During the analysis, the column temperature was maintained at 35 °C. The sample injection volume was 10 μL. The mobile phase flow rate was 0.4 mL/min. FB were eluted using a binary gradient composed of mobile phase A (water containing ammonium acetate, 1 mM, and acetic acid, 0.1%, *v*/*v*) and mobile phase B (methanol containing ammonium acetate, 1 mM, and acetic acid, 0.1%, *v*/*v*). Elution started with 99% mobile phase A which decreased linearly to 50% after 7 min and to 99% over the next 3 min. After 17 min, the column was re-equilibrated with 99% mobile phase A for 5 min. MS acquisition was performed in the selected ion recording (SIR) mode using a positive electrospray ionization mode (ESI+). The cone voltage was set at 20 eV. The *m*/*z* for FB1 and FB2/FB3 were 722.6 and 706.6, respectively. Data acquisition and instrument control were performed by MassLynx V4.1 (Waters Corporation).

### 2.3. Experimental Design and Sample Collection

The in vivo trial was carried out with the approval of the Institutional Animal Care and Ethics Committee of the University of Turin (Approval number: 319508/2017-PR). The study was conducted at the Department of Veterinary Sciences, following protocols previously described [39]. Briefly, 32 male ROSS 308 broiler chickens, 18 days old and weighing 751.9 ± 46.3 g, were housed in cages (2 birds/cage) in compliance with Directive 2007/43/EC and fed a standard basal diet. After a four-day acclimatization period (23 days old), birds were randomly allocated into four dietary treatment groups (*n* = 8 per group): K (basal diet only), OTA (basal diet + 0.26 ± 0.02 mg/kg OTA), ZEA (basal diet + 2.9 ± 0.2 mg/kg ZEA), and FB (basal diet + 33 ± 3 mg/kg FB1, 17 ± 2 mg/kg FB2, 11 ± 2 mg/kg FB3). The treatments were administered over a 10-day period, from day 23 to day 32 of age. At the end of the trial, blood samples were collected from the brachial vein, and all animals were euthanized using a sodium pentobarbital overdose. Liver and duodenum specimens were divided into aliquots and stored appropriately: a portion was frozen in liquid nitrogen for biochemical assays (lipid peroxidation, enzymatic activity, and glutathione—GSH-content) and the rest was placed in RNAlater^®^ solution (Sigma-Aldrich, Milan, Italy) for gene expression studies. Throughout the study, birds were monitored daily, with no mortality or clinical symptoms observed in any group. Tissue collection, sample processing, and all subsequent laboratory analyses were conducted by operators blinded to treatment allocation.

### 2.4. Growth Performance and Productive Parameters

Body weight (BW, g) was individually measured at the beginning (day 23) and the end of trial (day 32). The feed consumption was recorded at a cage level during the experimental period to calculate the overall average daily feed intake (ADFI, g/d) and the feed conversion ratio (FCR, g/g, DM basis) for each experimental group. Furthermore, based on ADFI and mean BW, the MYC intake per bird was calculated and expressed as µg MYC/kg BW/day.

### 2.5. Assessment of Serum Antioxidant Capacity (SAC)

The serum antioxidant capacity (SAC) was assayed following the manufacturer instructions through the OXY-Adsorbent test from Diacron (Grosseto, Italy), which determines the ability of serum (diluted 1:50 in water) to neutralize the oxidative activity induced by hypochlorous acid (HClO). The absorbance values were measured using the Glomax Multi detection system spectrophotometer (Promega, Madison, WI, USA) at 505 nm, and the results were expressed as mmol of HClO per mL.

### 2.6. Assessment of Lipid Peroxidation

Lipid peroxidation was determined by measuring the thiobarbituric acid reactive substances (TBARS) in hepatic supernatants, according to a modification of the method of Espín et al. [40], as already described [39]. Samples, standards, and blanks were mixed with the 2-thiobarbituric acid solution and incubated in a water bath at 95 °C for 60 min. After cooling and centrifugation, the formation of MDA-TBA adducts was measured in the clear supernatants at 532 nm. All samples were analyzed in triplicate, and results were expressed as nmol MDA per mg of tissue.

### 2.7. Total GSH Content and Enzymatic Activities

A portion of the liver tissue from each broiler was homogenized, and cytosolic fractions were isolated through differential ultracentrifugation [41]. The total GSH content in the cytosolic fractions was determined with Elmann’s reagent on trichloroacetic acid (TCA)-deproteinized samples, following previously established methods [42], and the results were expressed as μmol of GSH per gram of liver.

The activity of cytosolic total, μ-class, and α-class GSH S-transferases (GST) was measured as described in Cuccato et al., using 1-chloro-2,4-dinitrobenzene (CDNB), 3,4-dichloronitrobenzene (DCNB), and tert-butyl hydroperoxide (TBH) as substrates, respectively [43]. GSH peroxidase selenium-dependent (GPx) activity in the cytosolic fraction was measured via an indirect spectrophotometric assay, monitoring the rate of NADPH oxidation by GSH reductase [44], where the decrease in absorbance at 340 nm was directly proportional to the GPx activity. Additionally, NAD(P)H:quinone oxidoreductase 1 (NQO1) activity was measured by monitoring the reduction of 2,6-dichloroindophenol at 600 nm, using the continuous method by Lind et al. [45]. All enzymatic activities were assayed under Vmax conditions and were linear concerning both time and protein concentrations. Results of all the enzymatic assays were expressed as nmol/min per mg of protein.

### 2.8. Gene Expression Analysis: RNA Extraction, cDNA Synthesis, and qRT-PCR

Total RNA was isolated from liver and intestinal tissues using the Maxwell^®^ RSC simplyRNA Kit (Promega), according to the manufacturer’s protocol. RNA concentrations and purity were assessed with a NanoDrop ND-2000 spectrophotometer (Thermo Fisher Scientific, Waltham, MA, USA), and all samples exhibited A260/A280 ratios above 1.9. Complementary DNA (cDNA) was synthesized from 1 µg of total RNA using the iScript™ cDNA Synthesis Kit (Bio-Rad, Hercules, CA, USA), following the manufacturer’s instructions.

Primers were designed using Primer3 software (v3.0, Applied Biosystems, Foster City, CA, USA) based on *Gallus gallus* reference sequences from GenBank and Ensembl. Details of the primer sequences, annealing temperatures, and amplicon lengths are provided in Supplementary Table S1.

Quantitative real-time PCR (qRT-PCR) was carried out using the Bio-Rad CFX Opus 96 system with the following cycling conditions: 30 s at 95 °C, 40 cycles of 5 s at 95 °C, and 30 s at 60 °C. Briefly, to identify the most stable combination of internal control genes (ICGs) for each tissue type, the mRNA levels of a selected panel of candidate genes (i.e., *YWHAZ*, *GAPDH*, *HMBS*, *HPRT*, *GUSB*, *RPL13*, *RPL19*, *RPS7*, *SDHA*, *PGK2*, *VIM*, and *TFRC*) were quantified in chicken liver and duodenum samples. According to the analysis performed via NormFinder version 0.953, *YWHAZ*/*RPS7* (Stability value for best combination of two genes = 0.108) and *GUSB*/*HPRT* (Stability value for best combination of two genes = 0.030) were selected as ICGs for liver and duodenum, respectively. Gene expression levels were calculated using the ΔΔCt method and analyzed using the CFX Maestro software (v5.2.008.0222, Bio-Rad). FC values calculated for each gene are summarized in Supplementary Table S2.

### 2.9. Statistical Analysis

Data were expressed as mean ± standard error of the mean (SEM). The normality of the data distribution was rigorously assessed using the D’Agostino and Pearson omnibus test, with each experimental group comprising *n* = 8 individuals. The zootechnical measurements were recorded at the cage level (two birds per cage), thus in this case *n* is equal to four per group. To identify statistically significant differences among groups, a one-way analysis of variance (ANOVA) was employed, followed by Dunnett’s multiple comparison post hoc test. Statistical significance was defined as a two-sided *p*-value of <0.05. All analyses were performed using GraphPad Prism (version 7.03; GraphPad Software, San Diego, CA, USA).

## 3. Results

### 3.1. Effect on MYC on Growth Performance and Productive Parameters

The effects of OTA, ZEA, and FB on the growth performance of broilers during the 10 days of administration and the calculated daily MYC intake (µg MYC/kg BW/day) are shown in Table 1. No significant differences were recorded among the treatment groups.

### 3.2. Effect of MYC on Systemic and Hepatic OS Parameters

**Figure 1:**
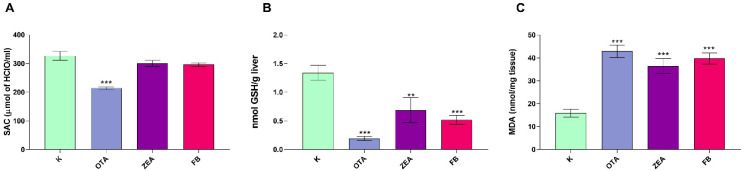
Effect of MYC exposure on the serum antioxidant capacity (SAC) (**A**), hepatic GSH content (**B**), and hepatic lipid peroxidation (**C**) in broilers after 10 days of exposure. K, Control group; OTA, Ochratoxin treated group; ZEA, Zearalenone treated group; FB, Fumonisin B treated group. Data are expressed as mean ± SEM, *n* = 8 (** *p* < 0.01 vs. K; *** *p* < 0.001 vs. K).

The SAC (Figure 1A) was significantly reduced in the OTA-treated group by approximately 30% compared to the control group (*p* < 0.001), while ZEA and FB exposure did not cause appreciable changes.

Exposure to MYC resulted in a pronounced OS in the liver, as demonstrated by the significant changes in GSH levels and lipid peroxidation parameters. There was a remarkable GSH depletion (Figure 1B) in all toxin-treated groups (*p* < 0.001), with OTA causing the greatest reduction. Concurrently, lipid peroxidation (Figure 1C), as measured via the TBARS assay, rose by about 3-fold vs. controls in all MYC-treated groups (*p* < 0.001).

### 3.3. Effect of MYC on Liver GSH—Dependent Enzymes and NQO1 Activity

GSH-dependent enzymes—including total GST, μ-class GST, selenium-independent GPx (α-class GST), and selenium-dependent GPx—and NQO1 activities were assayed in cytosolic fractions (Table 2). MYC exposure resulted in a general decrease in the tested parameters, which was most noticeable for total GST (*p* < 0.001), GPx-selenium dependent (*p* < 0.01 for OTA, and *p* < 0.001 for ZEA and FB), and NQO1 (*p* < 0.001). No significant differences were recorded among the treatment groups.

### 3.4. Effect of MYC on OS-Related Gene Expression in Liver and Duodenum

**Figure 2:**
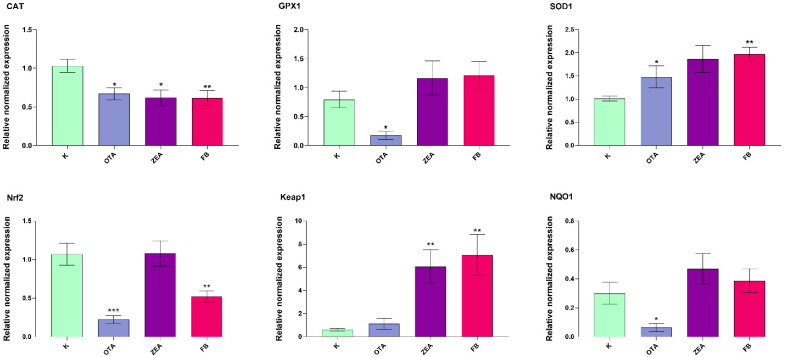
mRNA expression of the selected oxidative stress-related genes in the liver of broilers after 10 days of exposure, by means of qRT-PCR. K, Control group; OTA, Ochratoxin-treated group; ZEA, Zearalenone-treated group; FB, Fumonisin B-treated group. Data are expressed as mean ± SEM, *n* = 8 (* *p* < 0.05, ** *p* < 0.01, *** *p* < 0.001 vs. K).

**Figure 3:**
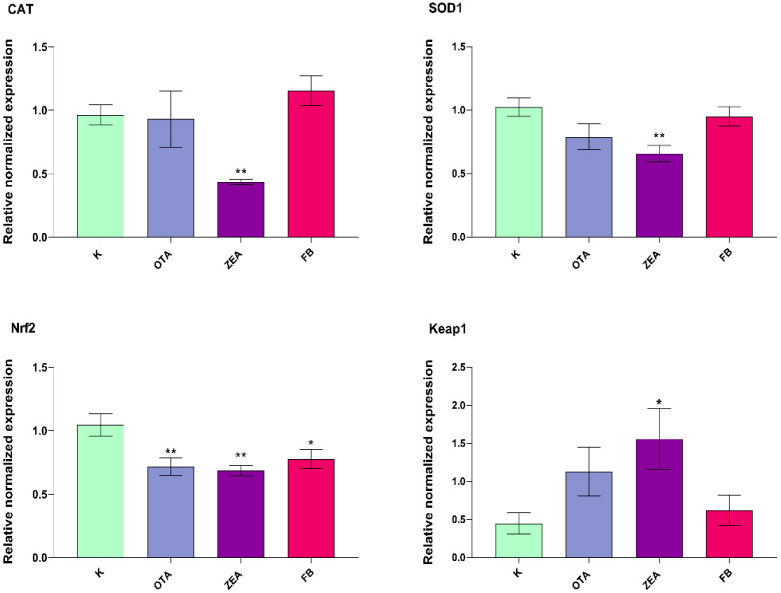
mRNA expression of the selected oxidative stress-related genes in the duodenum of broilers after 10 days of exposure, by means of qRT-PCR. K, Control group; OTA, Ochratoxin-treated group; ZEA, Zearalenone-treated group; FB, Fumonisin B-treated group. Data are expressed as mean ± SEM, *n* = 8 (* *p* < 0.05, ** *p* < 0.01 vs. K).

Relative mRNA expression levels of antioxidant defence genes in response to MYC exposure were analyzed in both the liver (*CAT*, *GPX1*, *SOD1*, *SOD2*, *Nrf2*, *Keap1*, and *NQO1*) and duodenum (*CAT*, *GPX1*, *SOD1*, *SOD2*, *Nrf2*, and *Keap1*). Among them, the significantly modulated ones are depicted in Figure 2 and Figure 3.

In the liver (Figure 2), *CAT* expression was significantly downregulated in all treatment groups compared to the control (*p* < 0.05 for OTA and ZEA, and *p* < 0.01 for FB). *GPX1* mRNA was reduced alike only by OTA (*p* < 0.05), while in ZEA and FB groups no significant changes were detected. In contrast, unlike OTA, ZEA (*p* < 0.05) and FB (*p* < 0.01) upregulated *SOD1* expression. Concerning the examined *Nrf2*/*Keap1* pathway components, only OTA (*p* < 0.001) and FB (*p* < 0.01) induced notable downregulation of *Nrf2*, whereas ZEA and FB significantly upregulated *Keap1* (*p* < 0.01). *NQO1* expression showed a significant decrease only in the OTA group (*p* < 0.05), with no significant alterations in the ZEA and FB groups.

In the intestinal tissue, the expression of the same set of genes involved in the antioxidant response showed a distinct pattern (Figure 3). *CAT* and *SOD1* expression levels were significantly decreased in the ZEA group only (*p* < 0.01), while OTA and FB had no significant effect. *Nrf2* expression was downregulated in all treatment groups (*p* < 0.01), whereas *Keap1* expression was significantly upregulated only by ZEA (*p* < 0.05), with no observed modulation for OTA and FB.

### 3.5. Effect of MYC on the Expression of Cytochrome P450s (CYPs) and 3β-Hydroxysteroid Dehydrogenase

The gene expression of phase I biotransformation enzymes, specifically CYPs, was assessed in both liver (*CYP2A6*, *CYP3A4*, *CYP1A5*, *CYP2C45*, *CYP2H1*) and intestinal tissues (*CYP1A4*, *CYP1A5*, *CYP3A5*).

**Figure 4:**
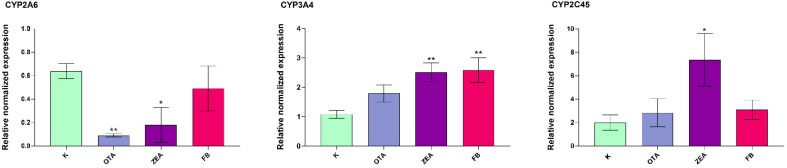
Cytochrome P450 gene expression in the liver of broilers after 10 days of exposure, by means of qRT-PCR. K, Control group; OTA, Ochratoxin-treated group; ZEA, Zearalenone-treated group; FB, Fumonisin B-treated group. Data are expressed as mean ± SEM, *n* = 8 (* *p* < 0.05, ** *p* < 0.01 vs. K).

In the liver, a significant gene modulation in response to the treatments was recorded only for *CYP2A6*, *CYP3A4*, and *CYP2C45* (Figure 4). *CYP2A6* was significantly downregulated by OTA (*p* < 0.01) and ZEA (*p* < 0.05), while *CYP3A4* expression was in general upregulated, although statistically significant changes were noticed only in the ZEA and FB groups (*p* < 0.001). Additionally, unlike OTA and FB, ZEA exposure markedly upregulated also *CYP2C45* (*p* < 0.05). The other examined CYP isoforms (*CYP1A5* and *CYP2H1*) remained unaffected. Furthermore, 3β-hydroxysteroid dehydrogenase (*3β-HSD*), an enzyme involved in the biotransformation of ZEA to β-zearalenol [46], showed a significant upregulation (*p* < 0.05) by approximately 3-fold in the ZEA-treated group (Table S2).

**Figure 5:**
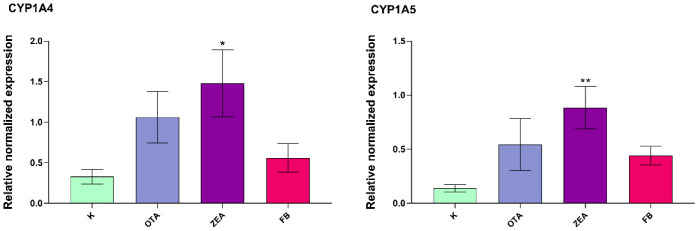
Cytochrome P450 gene expression in the duodenum of broilers after 10 days of exposure, by means of qRT-PCR. K, Control group; OTA, Ochratoxin-treated group; ZEA, Zearalenone-treated group; FB, Fumonisin B-treated group. Data are expressed as mean ± SEM, *n* = 8 (* *p* < 0.05, ** *p* < 0.01 vs. K).

Among the intestinal CYP isoforms analyzed (*CYP1A4*, *CYP1A5*, and *CYP3A5*), a significant upregulation was observed in *CYP1A4* (*p* < 0.05) and *CYP1A5* (*p* < 0.01) expression, particularly in response to ZEA exposure (Figure 5). The *CYP3A5* transcript levels remained unaffected.

### 3.6. Effect of MYC on the Expression of Hepatic and Intestinal Drug Transporters

**Figure 6:**
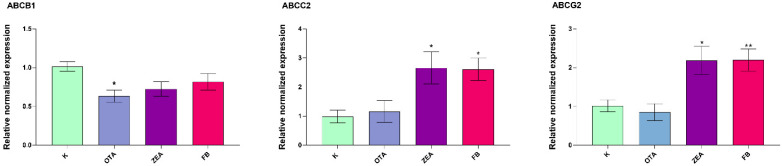
Gene expression of drug transporters in the liver of broilers after 10 days of exposure, by means of qRT-PCR. K, Control group; OTA, Ochratoxin-treated group; ZEA, Zearalenone-treated group; FB, Fumonisin B-treated group. Data are expressed as mean ± SEM, *n* = 8 (* *p* < 0.05, ** *p* < 0.01 vs. K).

The expression of ATP-binding cassette (ABC) family transporter genes (*ABCB1*, *ABCC2*, *ABCG2*) was evaluated in both hepatic and intestinal tissues of broilers following MYC exposure. In the liver (Figure 6), all the investigated transporters—*ABCB1*, *ABCC2*, and *ABCG2*— exhibited treatment-dependent modulation. *ABCC2* and *ABCG2* showed increased expression in response to FB (*p* < 0.01) and ZEA (*p* < 0.05), while OTA had no notable effect on either gene. In contrast, *ABCB1* expression was selectively reduced by OTA (*p* < 0.05).

**Figure 7:**
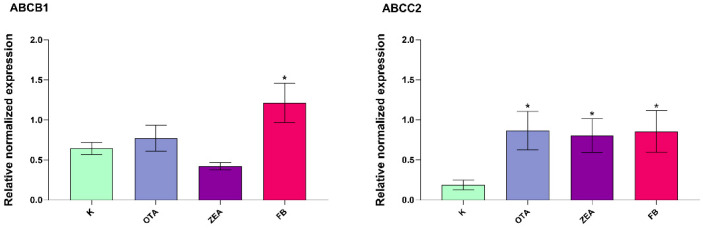
Gene expression of drug transporters in the duodenum of broilers after 10 days of exposure, by means of qRT-PCR. K, Control group; OTA, Ochratoxin-treated group; ZEA, Zearalenone-treated group; FB, Fumonisin B-treated group. Data are expressed as mean ± SEM, *n* = 8 (* *p* < 0.05 vs. K).

On the other hand, in the duodenum (Figure 7) there was a statistically significant increase in *ABCB1* expression in the FB group (*p* < 0.05), whereas *ABCC2* was remarkably induced by all MYC exposure (*p* < 0.05). No significant changes were observed in *ABCG2* expression in the intestinal samples.

## 4. Discussion

MYC feed contamination remains a persistent concern in poultry production due to its impact on animal health, productivity, and food safety. In the last decades, research has focused on the set up and validation of several MYDA to decrease MYC bioavailability, leading to “a reduction of mycotoxin uptake as well as distribution to the blood and target organs” [47]. Beside analytical determinations in tissues and/or excreta, several “biological endpoints”, including the effects on zootechnical performances, have been proposed to test the in vivo efficacy of MYDA [47].

In our study, growth performances of treated broilers resulted unaffected. This was expected for ZEA, for which no effects on these parameters are reported in the literature up to 800 mg/kg diet [10]. However, our findings are in contrast with previous reports describing significant impairments of zootechnical performances under comparable treatment periods and lower dosages for either FB [48] or OTA [49]. Among other factors, this discrepancy might be attributable to the older age (22 days) and, consequently, the higher average body weight (around 800 g at the beginning of the trial) of the birds from our study. Indeed, a later MYC exposure in the broiler production cycle appears to alter zootechnical parameters to a lesser extent compared to treatments involving much younger individuals [50]. This strengthened the need to investigate additional endpoints targeting more sensitive molecular parameters.

OS has been widely recognized as a common mechanism of MYC-induced toxicity [18]. From a systemic and hepatic OS perspective, OTA emerged as the most potent toxin among the three tested MYC. The inverse relationship between GSH depletion and rise in TBARS levels reflects the redox imbalance, contributing to oxidative damage. This observation aligns with the findings of Kövesi et al. [51], who reported similar effects in broilers following a 7-day exposure to 0.1 mg/kg feed OTA. Likewise, Li and colleagues observed an increased hepatic TBARS content (GSH not measured) using a lower OTA concentration (0.05 mg/kg) in broiler diet, but for a longer period (21 days) [52]. Overall, our results further confirm the ability of OTA to trigger both systemic and hepatic OS, even after short exposure periods and/or lower dietary concentrations.

In contrast, ZEA and FB altered the OS investigated parameters only at the hepatic level, suggesting a more localized oxidative injury. A comparable decrease in hepatic GSH was reported in broiler chickens exposed to 2.5 mg/kg ZEA in feed for a longer exposure (21 days), without concurrent increases in SAC or hepatic TBARS content [53]. Furthermore, when the feed ZEA content was reduced to 0.5 mg/kg and administered for 35 days, no OS endpoints (e.g., SAC, GSH, TBARS) were affected in broiler chickens [54]. As regards FB, evidence in the literature remains limited, as OS endpoints have been less investigated in chickens [54]. Increase in liver TBARS was not reported in chickens exposed to lower concentrations of FB (total FB levels of 10 mg/kg in feed) for 10 days [49]. By contrast, lipid peroxidation following the dietary exposure to FB1 (100 mg/kg) for 21 days or to total FB (600 mg/kg) for 10 days was previously described by Poersch et al. and Galli et al., respectively [55,56]. In line with our results, these findings suggest that even the short-term exposure to ZEA and FB, although less markedly than OTA, can perturb the hepatic oxidative balance with different mechanisms.

GSH-dependent antioxidant enzymes—GSTs and GPx—are pivotal in neutralizing peroxides and detoxifying electrophilic compounds [57,58]. The reduction in the hepatic GPx-selenium dependent activity upon OTA exposure has already been reported in broilers administered with higher dosages (from 0.05 mg/kg BW/day OTA by gavage to 1 mg/kg BW/day) or for longer time (up to 21 days) [22,49,52]. By contrast, the reduction in total GST and GPx-selenium dependent activities observed in the FB-treated group only partially agrees with previous reports. For instance, Poersch et al. and Fortuoso et al. found no statistically significant changes in hepatic GST activity in broilers dietary exposed to FB1 (100 mg/kg) for 21 days or FB (0.4 mg/kg) for 10 days, respectively [55,59]. Conversely, Galli et al. reported even an increase in hepatic GST activity following the exposure to 600 mg/kg FB in feed for 10 days [56]. Interestingly, Sousa et al. observed a decrease in GPx activity using lower FB concentrations (total FB levels of 5 and 10 mg/kg in feed) with the same 10-day exposure period [48]. Regarding the modulation of liver total GST and GPx-selenium dependent activities by ZEA, our findings are consistent with the reported reduction in GPx activity in broilers exposed to much higher doses of ZEA (2.5 mg/kg BW/day for 7 days) [60]. In contrast, no alterations in either liver GST or GPx activities were observed at a lower ZEA concentration (0.5 mg/kg in feed for 35 days) [54]. Taken together, these findings might point to a threshold for the sensitivity of these enzymes to ZEA.

NQO1 is an enzyme that contributes to the antioxidant defence, counteracting the activity of reactive metabolites [61]. To the best of our knowledge NQO1 has not been investigated so far in chickens exposed to the MYC tested in our study. However, the here-reported reduced activity may be associated with a raised susceptibility to OS.

The OS scenario induced by the examined MYC was also explored at gene expression level in the liver. Both OTA and FB exposure downregulated *Nrf2* expression, while ZEA and FB upregulated *Keap1*, suggesting the impairment of Nfr2 nuclear translocation and the subsequent inhibition of the antioxidant defence [62]. The observed OTA-induced response is consistent with the few available reports [51,52,63], whereas no data have been published about the effect of ZEA or FB on the Keap1-Nrf2 pathway in chickens. The resulting impairment in the transcription of downstream antioxidant genes may explain the consistent decline in enzyme activities, particularly for *GPx* and *NQO1*. The reduction in GPx-selenium dependent activity, especially in the OTA group, aligns with the observed downregulation of *GPX1* mRNA, suggesting a transcriptional suppression mechanism. The same trend of reduced enzyme activity and gene expression upon OTA treatment was observed in the already cited study by Li et al. [52]. By contrast, in our study the depression in GPx-selenium dependent activity in ZEA and FB groups was not matched by any transcriptional changes. Also, for NQO1, the enzyme activity reduction was reflected at transcriptional level only in the OTA group, fully in line with the relatively higher pro-oxidant effect of OTA compared to the other two MYC [64]. The discrepancy between the transcriptional and the enzymatic data in ZEA and FB groups is difficult to interpret. A possible explanation might be a posttranscriptional regulation by miRNA, as several studies demonstrated a relationship between such key-regulators and MYC exposure also in farm animals [65,66]. In the duodenum, the changes in antioxidant gene expression were less severe than in the liver, since a lower number of genes were affected by the treatments. Among the three tested MYC, ZEA had the strongest effect, suggesting that it may weaken the duodenum antioxidant defence by blocking the Nrf2 pathway, which is essential for the activation of protective genes [11,67].

CYP-dependent biotransformations are important determinants of the kinetics and the dynamics of a large array of drugs and toxicants, including some MYC. Only a limited number of investigations have addressed CYP modulation in response to the three MYC considered here. These studies either examined different CYP isoforms or, in the case of the intestine, focused on different segments [49,68]. As regards the liver, the exposure to OTA or ZEA resulted in a down-regulation of *CYP2A6*, particularly in the OTA group. In farm animals and humans, this subfamily is only marginally involved in the metabolism of drugs and toxicants [69] but participates in the biotransformation of several active principles of plant origin (e.g., coumarin, nicotine, flavones, flavonoids) [70]. Despite that *CYP2A6* in chicken liver is relatively less expressed than other isoforms (e.g., *CYP2C45* or *CYP3A4*) [71], its crucial role in the bioactivation of AFB1-to-AFB1 epoxide has been demonstrated in chickens [72,73] and other avian species [74]. In chick liver there was also a tendency to increase the expression of *CYP2C45* (ZEA only) and *CYP3A4* (ZEA and FB). It is worth noting that both families (particularly CYP3A) are deeply involved in the biotransformation of a large array of drugs, toxicants, feed additives, as well as endogenous substrates [71,75].

It has been long known that ZEA undergoes reductive biotransformations, mediated by α- and β-HSD and followed by conjugation [46]. More recently, the in vitro identification of several mono-hydroxylated (OH)-metabolites in humans and in several animal species [76,77], including chickens [78], pointed to the occurrence of an oxidative pathway. Interestingly, studies with in vitro systems expressing rat and human hepatic CYPs revealed the involvement of CYP3A and CYP2C subfamilies in the generation of the mentioned OH derivatives [76]. In keeping with our results, a time-dependent increase in *CYP3A*, *CYP2C*, and, to a lesser extent, *CYP2B* expression and functions were detected in liver from ZEA i.p.-dosed rats [79]. In the same animals a 2-fold elevation of liver *3β-HSD* expression was also reported, thereby matching what we found in ZEA-exposed chickens. In mammalian species pregnane X receptor (PXR) and constitutive androstane receptor (CAR) regulate the expression not only of the mentioned CYPs but also of other phase I (including HSD) and phase II enzymes, and a number of DTs [80,81]. Of note, although chickens lack both CAR and PXR and express only the closely related Chicken Xenobiotic Receptor (CXR) [82], the ZEA-mediated increase mainly in CYP3A and CYP2C was matched by the enhanced expression of both PXR and CAR in various rat and human hepatic cell systems [83,84].

In the only comparable study in chickens that could be retrieved), the dietary exposure to FB concentrations approaching the EU recommended levels for 15 days did not change the gene expression of *CYP1A* and *3A* subfamilies in the duodenum and in the liver [25]. The 3-fold increase in hepatic *CYP3A* transcripts observed in our study occurred after the exposure for a shorter period (10 days) but to approximately a double concentration of FB1, FB2, and FB3, which might at least partly explain the discrepancy with the results by Antonissen and colleagues [25]. In agreement with our data, a dose-related increase in the in vitro metabolism of two CYP3A substrates (erythromycin and ethylmorphine, [85]) was documented in liver microsomes from mallard ducks (*Anas platyrhyncos*) dosed with 5, 15, or 45 mg FB1/kg BW/day for 12 days [86].

*CYP1A*, *CYP2H*, and *CYP3A* are the most expressed CYPs in chicken intestine [87]. In our study only *CYP1A* appeared to be modulated by the MYC treatments, reaching the statistical significance for ZEA only. For comparison, in the cited study by Antonissen et al. [25], no changes in duodenal *CYP1A4*, *CYP1A5*, and *CYP3A37* were observed in FB-exposed chickens, a remarkable up-regulation being detected only in the jejunum. In keeping with the trend observed in the liver, ZEA appeared to behave as a CYP inducer also at enteric level, affecting the expression of both chicken *CYP1A* isoforms. A limited induction of the AhR-mediated pathway (*CYP1A1*, Aryl Hydrocarbon Receptor Nuclear Translocator, ARNT) has been reported in the liver from ZEA i.p.-dosed rats [79], but no information is available for the intestine. Enteric metabolism of ZEA has been well characterized in pig [88] and human enterocytes [77], resulting in the generation of α- and β-reduced derivatives and their corresponding phase II conjugated metabolites. In addition to what was found in the liver, our results point to the occurrence of a CYP-mediated ZEA oxidation also during the pre-systemic metabolism of the MYC. Interestingly, the chicken CYP1A subfamily is known to play opposite roles, i.e., detoxification vs. bioactivation, toward different MYC. In chicken liver microsomes CYP1A5 has been identified as the major isoform carrying out the hydroxylation of T-2 toxin yielding the less toxic 3′OH-T2 [26], while CYP1A1, the human ortholog of the avian CYP1A4, contributes significantly to the bioactivation of AFB1 to AFB1-epoxide [89].

The ATP-binding cassette (ABC) superfamily of DT includes P-glycoprotein (P-gp), the gene product of multidrug resistance 1 (*MDR1*, *ABCB1*), the multidrug resistance-associated protein 2 (*MRP2*, *ABCC2*), and the breast cancer resistance protein (*BCRP*, *ABCG2*). According to their expression in different tissues, they act mainly by preventing the entry of chemicals (e.g., skin, lungs, intestine, brain, blood vessels) or facilitating their excretion (e.g., liver, kidney) [90]. Therefore, they are crucial determinants of the absorption, distribution, and excretion and, hence, ultimately of the pharmaco-toxicologically effects of a broad range of drugs, feed additives, and toxicants. DT expression and regulation in different chicken tissues have been characterized [29,91,92,93], but very little is known about the nature of their substrates and the interaction with MYC in different tissues. Except for *ABCB1*, in our study there was an overall trend toward the increase in DT expression in both liver and intestine (duodenum), with FB showing the most consistent results (2- to 3-fold) in both tissues. In the mentioned report by Antonissen et al. [25], the exposure of broiler chickens to lower dietary FB concentrations (about half of those used in our trial) for a longer period (15 vs. 10 days) triggered the rise in *ABCB1* and *ABCC2* in the jejunum but not in the liver. Interestingly, they observed a slight decrease in the Area Under Curve (AUC) of oral enrofloxacin, pointing to a lower absorption rate of the fluoroquinolone when orally co-administered with FB.

## 5. Conclusions

Overall, the findings of our study demonstrated that all the investigated MYC (i.e., OTA, ZEA, and FB) disrupted the chicken antioxidant defence even after a short exposure period at feed contamination levels 2- to 3-fold higher than the recommended EU limits. Such effect is most pronounced and generalized (i.e., impairment of SAC) in the case of OTA exposure compared to ZEA and FB, suggesting a highest pro-oxidant activity by OTA. In addition to redox imbalance, all three MYC modulated the hepatic and duodenal expression of genes involved in both xenobiotic metabolism and transport (i.e., some CYP isoforms and DT), with ZEA being more effective in modifying the CYP and FB the DT mRNA levels, respectively. Although the transcriptional changes need to be further validated at protein level, they point to the potential impact of MYC exposure on drug kinetics, which might negatively affect drug efficacy and/or residue formation and persistence in chickens. Further in vivo studies are needed to confirm our results under field conditions.

Finally, our findings need to be considered in the light of some limitations. The experimental design did not include a dose–response schedule. Moreover, the selected MYC feed concentrations were 2 to 3-fold higher than the maximum levels currently recommended by the EU, with the aim to maximize the potential effects of the tested MYC on the selected endpoints. However, for several of the investigated parameters, published data are still limited or related to considerably higher doses or longer exposure times. This makes our findings particularly relevant, as they provide novel information in a concentration range close to field contamination levels detected in some non-EU countries. Moreover, results from gene expression analysis should be partially (OS pathways) or totally (CYPs and DT) confirmed at protein level.

Taken together, data generated from this study are expected to serve as key toxicological endpoints in upcoming studies aimed at testing the efficacy of two innovative smectite-based MYDA: SeOX, a tri-octahedral smectite combined with lignocellulose, and CHS, a di-octahedral smectite functionalized with a non-toxic organic modifier [94].

## Figures and Tables

**Table 1:** Growth performance and productive parameters of broiler chicken fed experimental diets.

	K	OTA	ZEA	FB	*p* Value
Initial body weight (d23) (g)	1011 ± 10	1004 ± 12	996 ± 11	993 ±11	0.509
Final body weight (d32) (g)	1944 ± 18	1968 ± 14	1959 ± 22	1965 ± 10	0.407
ADFI 23–32 days of age (g)	143 ± 2.6	145 ± 4.8	149 ± 3.9	144 ± 1.3	0.647
FCR 23–32 days of age	1.53 ± 0.01	1.51 ± 0.2	1.53 ± 0.03	1.49 ± 0.1	0.951
MYC intake (µg MYC/kg BW/day)	-	0.025 ± 0.002	0.293 ± 0.017	5.967 ± 0.128	-

K, Control group; OTA, Ochratoxin-treated group; ZEA, Zearalenone-treated group; FB, Fumonisin B-treated group. Data are expressed as mean ± SEM (*n* = 4).

**Table 2:** Enzyme activities of total GST, μ-class GST, selenium independent and dependent GPx, and NQO1 in liver samples from treated broilers.

Parameter	Treatments			
	K	OTA	ZEA	FB
Total GST (nmol/min/mg protein)	523.8 ± 27.66	382.5 ± 18.03 ***	365.9 ± 16.77 ***	297.1 ± 20.23 ***
µ-class GST (nmol/min/mg protein)	0.880 ± 0.10	0.643 ± 0.09	0.614 ± 0.09	0.732 ± 0.12
GPx-selenium independent (α-class GST)	17.00 ± 2.32	13.36 ± 3.49	9.61 ± 5.00	8.71 ± 3.03
GPx-selenium dependent	45.37 ±1.35	35.33 ± 2.02 **	35.35 ± 1.76 **	30.58 ± 2.10 ***
NQO1 (nmol/min/mg protein)	367.5 ± 14.69	274.9 ± 17.27 ***	234.6 ± 16.99 ***	204.9 ± 7.40 ***

K, control group; OTA, Ochratoxin-treated group; ZEA, Zearalenone-treated group; FB, Fumonisin B-treated group. Data are expressed as mean ± SEM, *n* = 8 (** *p* <0.01, *** *p* <0.001 vs. K).

## Data Availability

The original contributions presented in the study are included in the article/Supplementary Materials, and further inquiries can be directed to the corresponding author.

## Supplementary Materials

The following supporting information can be downloaded at https://www.mdpi.com/article/10.3390/foods14244249/s1. Table S1. Primers for Quantitative Real-Time PCR (qRT-PCR). Table S2. Fold-change (FC) values of the genes investigated by Quantitative Real-Time PCR (qRT-PCR).
