# Supplementary material for: Short-Term Dietary Exposure to Ochratoxin A, Zearalenone or Fumonisins in Broiler Chickens: Effects on Cytochrome P450 Enzymes, Drug Transporters and Antioxidant Defence Systems
Source: Foods. 2025 Dec 10;14(24):4249. doi: 10.3390/foods14244249 (PMC12732176; doi:10.3390/foods14244249)
Supplement: Supplementary file 1 [file foods-14-04249-s001.zip › Supplementary Table S1.pdf]

Table S1. Primers for Quantitative Real-Time PCR (qRT-PCR).

Gene	Accession no.	Sequence	Melting temperature (°C)	Amplicon size (bp)
CYP2A6	KX687985	FW: CCCTCTCCTAAACAGATGCG	57.39	
		REV: TTGCTGTCTCCCATCCTGC	58.81	149
CYP2H1	NM_001001616.1	FW: TCCTTCCCCTTAATGTTCCACA	61.08	
		REV: GGGAGACAGCAAAGGGAATATC	59.61	98
CYP3A4	NM_001329508.2	FW: TGGTAGTCATGATCCCAGCC	59.81	113
		REV: GGGGTCAATGTTCTCTCCGT	58.03	
CYP3A5	NM_001001751.2	FW:CCAATAAGGCTCCGCTCAC	58.08	110
		REV: GGTTCTCTCAAGCCGTCCT	56.35	
CYP2C45	NM_001001752.2	FW:AGAGCGACTTCTTCATTCCCT	57.43	95
		REV: GATGGCGGTCAGGAGTAAGA	57.4	
CYP1A4	NM_205147.1	FW:GATGTCCGCGTCCAACCC	61.32	86
		REV: GCGGTTGTCACGGTGTCAA	60.47	
CYP1A5	NM_205146.2	FW:CGCAGATCCCAAACGAGAAG	60.61	
		REV: GCGGTTGTCACGGTGTCAA	60.47	76
3-beta HSD	NM_205118	FW:GGCTGCTGGACAAAGACTTC	57.17	
		REV: GCCCAAGGTGTCAATGATGG	60.67	133
Nrf2	NM_205117	FW: AATCAAACTCAGCCACCCAG	57.35	
		REV: CAGCCAGGTTGTCGTTTTCA	59.3	142
CAT	NM_001031215.2	FW: GGCACTGCTGGACAAATACA	56.66	
		REV: AAGTGGCTTGCGTGTATGTC	55.94	71
GPX1	NM_001277853.2	FW: TTCGGGCACCAGGAGAACGC	67.68	91
		REV: TGGTGAAGTTGGGTTTGAAGC	60.04	
SOD1	NM_205064.1	FW: GGGAGGAGTGGCAGAAGTAG	56.2	115
		REV: CCCTCTACCCAGGTCATCAC	56.35	
SOD2	NM_204211.1	FW: GGAGCAGGGACGTCTACAAA	57.54	81
		REV: CCCAGCAATGGAATGAGACC	59.95	
**Keap 1**	XM_025145847.1	FW: ACTTCGCTGAGGTCTCCAAG	56.75	
	**Li et al., 2020**	RW: CAGTCGTACTGCACCCAGTT	55.65	142
NQO1	NM_001277619	FW: CATCTTCCAGTTCCCACTGC	57.58	
		RW: GCTGTACATGGAGCCCATCC	59.97	178
ABCB1	NM_204894.1	FW: ACAACAGTCGGGAGGTGTC	54.62	123
		REV: GCTGTGTTCCCTTGTCTCCT	56.32	
ABCC2	XM_015288821.2	FW: TCCTTGTTCTTTGTCACCACA	59.22	122
		REV: AGTAGGCAGACACGCGATAA	58.39	
ABCG2	NM_001328490.1	FW: TCCTTGTTCTTTGTCACCACA	56.08	124
		REV: AGTAGGCAGACACGCGATAA	56.02	
RPS7	XM_001234708.4	FW: GCCCAAGCCAACGAGAAAA	61.0	138

		REV: TTTACGCGGATTCTCTTGCC	60.48	
YWAHZ	NM_001031343.1	FW:ACAAAGACAGCACGCTAATAATG	57.27	
		REV: ATTCTCCCCTCCTTCTCCTG	57.18	104
GUSB	NM_001039316.2	FW:TGATTGGGGAACTCATCTGGA	59.94	
		REV: CGTTGGCGGGTAAATATTCCT	61.0	97
HPRT	NM_204848.1	FW:ACGTTGCTGTCTCTACTTAAGC	53.98	
		REV: CCCACACTTCGAGGAGTTCT	56.36	86

Italicized gene names indicate the internal control genes used as references.
