# Supplementary material for: Short-Term Dietary Exposure to Ochratoxin A, Zearalenone or Fumonisins in Broiler Chickens: Effects on Cytochrome P450 Enzymes, Drug Transporters and Antioxidant Defence Systems
Source: Foods. 2025 Dec 10;14(24):4249. doi: 10.3390/foods14244249 (PMC12732176; doi:10.3390/foods14244249)
Supplement: Supplementary file 1 [file foods-14-04249-s001.zip › Supplementary Table S2.pdf]

Table S2. Fold-change (FC) values of the genes investigated by Quantitative Real-Time PCR (qRT-PCR).

Gene	Organ	Expression values				FC values		
		**K**	**OTA**	**ZEA**	**FB**	**OTA**	**ZEA**	**FB**
CAT	Liver	1.0291	0.6689	0.6188	0.6130	0.65	0.60	0.60
GPX1	Liver	0.7928	0.1724	1.1637	1.2092	0.22	1.47	1.53
SOD1	Liver	1.0103	1.4781	1.8668	1.9679	1.46	1.85	1.95
SOD2	Liver	1.0075	0.9091	0.7443	0.7462	0.90	0.74	0.74
Nrf2	Liver	1.0716	0.2248	1.0792	0.5236	0.21	1.01	0.49
Keap1	Liver	0.6288	1.1267	6.0715	7.0771	1.79	9.66	11.25
NQO1	Liver	0.3006	0.0641	0.4713	0.3883	0.21	1.57	1.29
CYP2A6	Liver	0.6387	0.0898	0.1782	0.4911	0.14	0.28	0.77
CYP3A4	Liver	1.0755	1.7866	2.5051	2.5768	1.66	2.33	2.40
CYP1A5	Liver	1.3804	1.7871	1.7804	3.6762	1.29	1.29	2.66
CYP2C45	Liver	2.0079	2.8320	7.3510	3.0983	1.41	3.66	1.54
CYP2H1	Liver	0.7286	0.8342	0.4521	0.9106	1.14	0.62	1.25
ABCB1	Liver	1.0153	0.6342	0.7243	0.8176	0.62	0.71	0.81
ABCC2	Liver	0.9873	1.1616	2.6559	2.6070	1.18	2.69	2.64
ABCG2	Liver	1.0161	0.8500	1.9837	2.1993	0.84	1.95	2.16
3b-HSD	Liver	0.6608	/	1.6090	/	/	2.43	/
CAT	Duodenum	0.9636	0.9307	0.4341	1.1535	0.97	0.45	1.20
GPX1	Duodenum	0.7680	0.7047	0.6355	0.4441	0.92	0.83	0.58
SOD1	Duodenum	1.0243	0.7902	0.6583	0.9513	0.77	0.64	0.93
SOD2	Duodenum	1.0087	1.1177	0.9701	1.0474	1.11	0.96	1.04
Nrf2	Duodenum	1.0446	0.7160	0.6859	0.7770	0.69	0.66	0.74
Keap1	Duodenum	0.4507	1.1300	1.5598	0.6213	2.51	3.46	1.38
CYP1A4	Duodenum	0.3296	1.0623	1.4798	0.5621	3.22	4.49	1.71
CYP1A5	Duodenum	0.1404	0.5441	0.8855	0.4420	3.87	6.31	3.15
CYP3A5	Duodenum	0.3374	0.3167	0.1666	0.7022	0.94	0.49	2.08
ABCB1	Duodenum	0.6463	0.7741	0.4229	1.2161	1.20	0.65	1.88
ABCC2	Duodenum	0.1912	0.8689	0.8055	0.8578	4.54	4.21	4.49
ABCG2	Duodenum	1.0527	1.0234	0.7241	1.0433	0.99	0.69	0.99

Statistically significant $p < 0.05$
